# Problematic Internet Pornography Use and Psychological Distress among Emerging Adults in Malaysia: Gender as a Moderator

**DOI:** 10.3390/ijerph19063682

**Published:** 2022-03-19

**Authors:** Soon-Aun Tan, Yee Shan Goh, Norzarina Mohd Zaharim, Su Wan Gan, Chin Choo Yap, Sarvarubini Nainee, Ling Khai Lee

**Affiliations:** 1 Department of Psychology and Counselling, Faculty of Arts and Social Science, Universiti Tunku Abdul Rahman, Jalan Universiti, Bandar Barat, Kampar 31900, Malaysia; yyshan133@gmail.com (Y.S.G.); swgan@utar.edu.my (S.W.G.); sarvarubini@utar.edu.my (S.N.); 2 School of Social Sciences, Universiti Sains Malaysia, Gelugor 11800, Malaysia; norzarina@usm.my; 3 Department of Psychology, School of Medical and Life Sciences, Sunway University, No 5, Jalan Universiti, Bandar Sunway 47500, Malaysia; gracey@sunway.edu.my; 4 Department of Languages and Linguistics, Faculty of Arts and Social Science, Universiti Tunku Abdul Rahman, Jalan Universiti, Bandar Barat, Kampar 31900, Malaysia; leelk@utar.edu.my

**Keywords:** internet pornography, psychological distress, emerging adults, Malaysia, gender

## Abstract

Internet pornography use (IPU) refers to Internet-based sexually explicit materials that are ultimately used to elicit sexual feelings or thoughts. The accessibility of Internet pornography could lead to excessive exposure to pornographic messages, posing a risk to heavy users’ psychological health. This paper offers a preliminary understanding of the relationship between Internet pornography use and psychological distress among emerging adults and the moderating role of gender in the association. This cross-sectional study has taken a purposive sampling approach to recruit 144 emerging adult pornography users via the online survey method. The results indicated that males reported having more problematic Internet pornography use, and there were no gender differences in psychological distress. Meanwhile, gender is a significant moderator between Internet pornography use and psychological distress. The females were found to be more psychologically affected by their problematic Internet pornography use than the males. Overall, this study has provided a novel finding of the moderating role of gender in problematic Internet pornography use and psychological distress in the Malaysian context. This study also calls for a gender-focused sexual health programme for Malaysian emerging adults. Furthermore, the scores of problematic IPU in this study raise a concern over the effectiveness of current sex education in Malaysia. The scores may highlight the need to provide education targeting Internet pornography use.

## 1. Introduction

Over the past decade, the advent of the high-speed Internet has made sexual content easily accessible [1], where Internet pornography use (IPU) has become more widespread [2,3,4]. Furthermore, since the outbreak of the COVID-19 pandemic, people have spent more time staying indoors, which has changed the way they express their sexuality [5], especially over the Internet. As a result, the global usage of Internet pornography has spiked up drastically [6], particularly during the pandemic. Evidence shows that an average of 130 million people were reported to have visited Pornhub, a famous global porn site [7], on a daily basis, compared to a daily average of 115 million before the pandemic [8]. Scientific studies have shown that problematic IPU is associated with various psychological distresses [9,10] among males and females, inclusive of guilt and shame [11], stress [12,13], and relationship conflicts [14]. However, the association between problematic IPU and psychological distress among emerging adults in Malaysia is still unclear.

Little knowledge about the moderating role of gender in this relationship is known. Emerging adulthood involves an individual’s developmental stage between late adolescence and early adulthood. It is often associated with substantial life changes, such as increased exploration of sexuality [15,16]. Literature shows that more emerging adult men than women have reported watching pornography [4,16]. However, the investigation into the effects of pornography use yielded mixed findings for both men and women.

To date, there is still a lack of consensus on the definition of problematic IPU. As such, several terms have been used in other literature, such as excessive use of pornography [17], problematic online pornography use [18], pornography addiction [19], and cybersex addiction [2]. Nonetheless, the term “problematic Internet pornography use” (IPU) will be used in this paper.

### 1.1. Pornography Use in Malaysia

Pornography use is deemed illegal in a sexually conservative country such as Malaysia. However, on average, three to four out of every ten Malaysians have been found subscribing to, or viewing, pornography weekly [20]. A recent study also reported a 74.5% lifetime prevalence rate of IPU among college students in [21]. Based on the report presented by Pornhub, Malaysia is ranked fourth globally in online searching for the keyword “coronavirus”. However, the reason behind seeking further information on the coronavirus on Pornhub is unknown [22]. Besides censoring relevant pornographic materials, the Malaysian Communications and Multimedia Commission (MCMC) [23] has actively and efficiently banned Malaysians from accessing almost 2500 pornography sites [24]. Moreover, Section 292 of the Penal Code states that owning any form of Internet pornographic material is illegal in this country [25].

Thus far, studies in Malaysia have consistently examined pornography use among teenagers and adolescents. For instance, past studies have associated pornography use with teenage pregnancies [26], premarital sex [27], and the legal perspective on regulating child pornography [28]. Meanwhile, the study examining pornography use among emerging adults in Malaysia remains scarce. It is thus imperative to explore the problematic IPU of emerging adults as the usage of pornography has heightened sexual risks and encouraged sexual experimentation [15,16].

### 1.2. Problematic IPU and Psychological Distress

As problematic IPU has become more prevalent, researchers have begun to study its potential connection with psychological distress. Common psychological distresses is found concurrently and over time included anxiety, stress, anger, and depressive symptoms [9]. Problematic IPU also negatively impacts identity development and self-worth [29]. It also affected the users’ self-esteem, reduced their efficacy at work, and created a sense of dissociation from the real world [30]. Meanwhile, qualitative studies conducted among problematic Internet pornography users have also documented the close association between problematic IPU and psychological distresses, such as guilt, shame, disgust, and dissatisfaction with their usage [11]. Although this qualitative study provides an in-depth understanding of the psychological distresses experienced through IPU, the study only focuses on males, where readers have a limited understanding of whether similar distresses are also felt by females.

Despite these harmful effects, other studies have suggested some potential positive gains of IPU in that it enhances sexual knowledge [31,32], openness [33], and yields more significant positive effects in men’s lives [34]. The act of watching porn is also categorised as an emotional coping mechanism (e.g., stress relief and relaxation) [35]. However, these individuals can end up experiencing more issues related to problematic IPU [36]. Although the positive impact of IPU still requires more studies in various cultural contexts, these findings cast doubt on the assumption that IPU is exclusively harmful to individuals. While the Western literature showed problematic IPU affecting psychological distress, no local study illustrated this link in the Malaysian context. Hence, the present study intends to provide local evidence for the link.

### 1.3. Gender Differences in Problematic IPU

The after-effect of pornography usage may vary according to the personal threshold. Some individuals are more susceptible to the effects of pornography than others. In terms of gender, the existing literature suggests that a gender gap exists, with males often watching more pornography than females [3,9,16,37,38]. It is speculated that men without a stable partner are more inclined to turn to pornography to fulfil their sexual desires [39]. Males were found to use Internet pornography more frequently than females during the pandemic [40].

Furthermore, male pornography users are more likely to meet the criteria of cybersex addiction, demonstrated addiction, and compulsive sexual behaviour [2]. Past studies also found that males tend to report higher problematic pornography use [41,42,43,44] and compulsive IPU [45] than their female counterparts. Another longitudinal study by Bőthe et al. [43] found that problematic pornography use level were significantly increased for girls, while a slight decrease was observed in boys in a comparison of before and during the pandemic. However, girls were reported to have lower problematic pornography use than boys.

### 1.4. Gender as a Moderator

Traditionally, the opponents of IPU claimed that it resulted in risky sexual behaviour [16] and impacted mental health [46]. For instance, pornography use among males is more likely to result in a greater level of depression [47] and reported more adverse effects of IPU [48] than females. However, proponents of IPU claim that the positive effects of IPU on both genders are evident. Studies have illustrated that IPU can improve sexual satisfaction, enhance sexual knowledge, and relieve boredom among men [49]. Similarly, women reported a greater positive effects in their lives associated with pornography use [48].

The inconsistencies in the gender difference in the association between pornography use and psychological distress may suggest that a moderating variable such as gender can explain the linkage better. Although studies have consistently illustrated a gender difference in pornography use, further explanation deserves equal attention on which gender is more susceptible to psychological distress in the context of problematic IPU. Most of the commercial pornographic contents are made targeting male users [50]. These contents presented by the mainstream media often devalue and objectify females but prioritise males’ enjoyment [51]. Engle [52] claimed that it was common to see unrealistic scenes containing devaluation and objectification of females as Internet pornography (IP) production dominates the male industry. Thus, males and females often encounter different experiences after watching IP.

Theoretically, the present study is guided by the sexual script theory [53], which explains a set of scripts that govern individuals’ sexual behaviour. The sexual scripts are gender-specific, where the expectations of sexual behaviour differ between males and females [54]. Continuous exposure to IP shapes the scripting process [55], where the sexually explicit messages and sexual behaviour portrayed in the IP can influence the perception, affection, cognition, and behavioural aspects of sexuality [48]. The sexual scripts theory varies across cultural contexts. However, to date, the studies still centralise on the Western samples, leaving a paucity in the Eastern samples.

Although the literature suggests that there are links between problematic IPU and psychological distress, these links are often moderated by other variables, such as experiential avoidance [56], relationship satisfaction, and sexual satisfaction [57]. Furthermore, years of pornography studies have illustrated consistent differences in the usage of pornography by both sexes, where males often watch more IP and experience greater psychological distress than females [3,4,16,37,38,47]. However, amidst inconsistencies in the effect of pornography use and psychological distress, it is not clear whether an individual factor such as gender can better explain the difference in such an association.

The existing literature on the role of gender on psychological distress concerning problematic IPU is scarce. However, past studies found that gender plays a significant moderating role in the relationship between an individual’s addiction to internet content. The evidence showed gender differences in problematic social media use (i.e., Instagram) and mental health [58]. Yurdagül et al.’s [58] study on problematic Instagram use and psychopathological consequences indicated that gender moderated the link, whereby problematic Instagram use was associated with loneliness in general and social anxiety among males. Thus, it is believed that gender differences may exist in the problematic usage of Internet pornographic content and psychological distress among Malaysian emerging adults.

## 2. The Present Study

In the present study, we attempted to expand the literature on problematic IPU. First, we targeted emerging adults as survey participants aged between 18 and 29. The sampling practise is different from other studies on IPU in Malaysia. It primarily focuses on teenagers [59,60,61]. As such, the present study would fill in the knowledge gap about the problematic IPU of emerging adults. The knowledge of gender differences in problematic IPU is scarce, particularly in the Malaysian context. Moreover, the moderating role of gender remains underreported even when the pornography literature notes gender differences, especially in the Western context [37,62,63,64]. Thus, the present study took a step further by examining the moderating role of gender on problematic IPU and psychological distress among emerging adults in Malaysia. The research questions are the following:

1. Is there any gender difference in problematic IPU (salience, mood modification, conflict, tolerance, relapse, and withdrawal) among emerging adults in Malaysia?
2. Is there any association between problematic IPU and psychological distress among emerging adults in Malaysia?
3. Does gender moderate the association between problematic IPU and psychological distress among emerging adults in Malaysia?

## 3. Methods

### 3.1. Participants

A total of 144 Malaysian self-identified Internet pornography users participated in the present study. The mean age of the participants was 21.41 years, with a standard deviation of 3.49. More than half of the participants were men (63.2%). About 68% of the participants were Chinese, followed by Malays (19.4%), Indians (11.1%), and other ethnicities (1.4%). 68.1% are students, 23.6% are full-time employees, and 8.3% are unemployed. More than half of the respondents were single (58.7%), 11.2% were married, and 30.1% were currently in a relationship (see Table 1).

### 3.2. Research Design and Procedure

This study employed a correlational and cross-sectional quantitative research design. Samples were recruited using the purposive sampling method, with participants who were self-identified Internet pornography users and Malaysian emerging adults aged between 18 and 29. Data collection was performed via Qualtrics survey, an online survey platform. Researchers posted the online survey link and a description of the project on social media accounts such as Facebook, Instagram, and LinkedIn. An information sheet was attached to the first page of the survey. The first page entails the objectives of the study, participants’ rights, risks and benefits of participation, and the policy of privacy and confidentiality of participants. The participants were then requested to provide their consent before answering the questions. The Institutional Research Ethics Committee has reviewed and approved the research procedure.

### 3.3. Measures

Kessler Psychological Distress Scale (K-6) [65] is a self-administered global measure of distress relevant to depressive and anxiety symptoms over four weeks. This inventory consists of six items. The items are rated on a 5-point Likert scale from 1 (none of the time) to 5 (all of the time). A mean score was computed with a higher score corresponding to higher psychological distress. The Cronbach Alpha value reported at 0.86 indicates excellent reliability.

Problematic Pornography Consumption Scale [42] consisting of 18 items measures participants’ experiences with pornography usage. Participants must respond to each statement using a 7-point Likert scale ranging from 1 (Never) to 7 (All the time). This scale consists of the following six factors (with three items for each): salience, mood modification, conflict, tolerance, relapse, and withdrawal. The mean score was computed with a higher score corresponding to more problematic IPU. The coefficient ranging between 0.70 and 0.95 indicated acceptable reliability for the total scale and subscales of the present study.

### 3.4. Data Processing and Plan of Analysis

The data was processed and analysed using IBM SPSS version 23. Several data analysis techniques were used. Firstly, an independent-sample *t*-test was used to examine gender differences in overall and sub-dimensions of problematic IPU. Next, Pearson product-moment correlation analysis was used to determine the association between problematic IPU and psychological distress. Lastly, the SPSS Macro PROCESS [66] Model 1 (i.e., simple moderation model) was utilised to test the moderating effect of gender in the relationship between problematic IPU and psychological distress. A simple slope graph was plotted based on the analysis output of SPSS Macro PROCESS.

## 4. Result

### 4.1. Gender Differences in Problematic Internet Pornography Use and Psychological Distress

Gender differences in problematic IPU and psychological distress among emerging adults were examined using an independent sample t-test (Table 2). Results revealed significant gender differences in problematic IPU for the whole scale and sub-scales except for the withdrawal subscale (*t* = 1.81, *p* = 0.073). The significant gender differences postulate that males tend to experience higher problematic IPU than females (Table 1).

### 4.2. Correlation between Problematic Internet Pornography Use and Psychological Distress

A significant correlation was found between problematic IPU and psychological distress, *r*(142) = 0.20, *p* = 0.007 (Table 3). Meanwhile, correlations between the sub-scales of problematic IPU and psychological distress were significant except for salience, *r*(142) = 0.13, *p* = 0.061, and tolerance, *r*(142) = 0.12, *p* = 0.076.

### 4.3. Moderating Effect of Gender

Hayes’s SPSS Macro PROCESS analysis [66] Model 1 was used to examine the moderating effect of gender in the association between problematic IPU and psychological distress. The overall model in psychological distress was statistically significant, *F*(3, 140) = 5.02, *p* = 0.002, R^2^ = 0.097. The R^2^ change of 0.042, *p* = 0.011, indicated the additional of interaction term of gender in the model was significant. Psychological distress was significantly and positively associated with problematic IPU, *β* = 0.36, *t*(140) = 3.75, *p* < 0.001, 95% CI [0.15, 0.51]. Gender was not significantly linked to psychological distress, *β* = 0.53, *t*(140) = 1.62, *p* = 0.108, 95% CI [0.11, −0.12]. The interaction between problematic IPU and gender on psychological distress was found to be significant *β* = −0.30, *t*(140) = −2.56, *p* = 0.011, 95% CI [−0.54, −0.07]. Therefore, it can be concluded that gender moderated the effects of problematic IPU on psychological distress.

**Figure 1:**
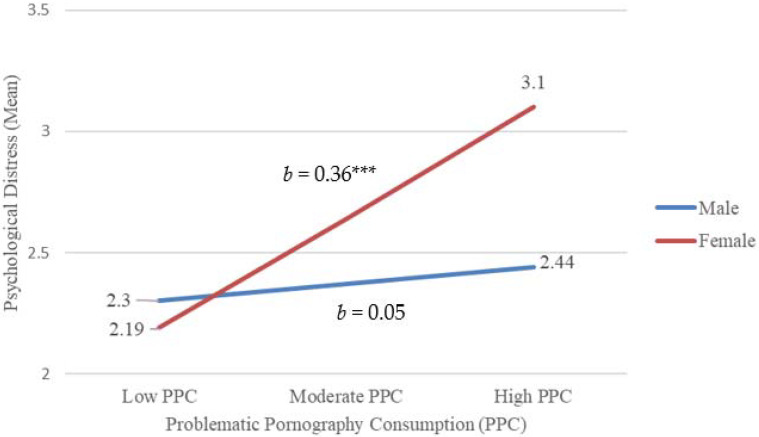
Interaction between problematic internet pornography use and gender in predicting psychological distress (*n* = 144). Note: *** *p* < 0.001.

Figure 1 shows the interaction plot. The standardized slope for problematic IPU was significant for females (*β* = 0.36, *t*(140) = 3.75, *p* < 0.001, 95% CI [0.17, 0.54]) but not for males (*β* = 0.05, *t*(140) = 0.75, *p* = 0.46, 95% CI [−0.09, 0.19]). At a low problematic IPU, females showed fewer psychological distress symptoms than males. While at a higher problematic IPU, females tended to report higher psychological distress than males. Consequently, the moderating effect indicated that the degree of problematic IPU had a more positive effect on females’ psychological distress than male emerging adults.

## 5. Discussion

The present study assessed the gender difference in problematic IPU and psychological distress and the moderating role of gender between problematic IPU and psychological distress among emerging adults in Malaysia. This study further confirmed and extended the existing knowledge that there is persistence in gender on problematic IPU and psychological distress. In line with a previous study on self-reported of IPU using Western samples [2], males were more likely to report problematic IPU. Interestingly, compared to Malaysia, these samples come from participants who are more liberal and well-educated in sex education, where IPU is widely accepted such as the United States. This shows that although Malaysia’s legal, education, and media greatly condemn IPU and highlight the negative consequences of IPU, this does not prevent them from the IPU, particularly among males. It could be noted that males’ IPU is regarded as a socially acceptable behaviour that can help to gain sexual knowledge [31,48]. Therefore, they are less likely to receive criticism for watching pornography, further escalating their IPU consumption to a problematic stage.

However, the findings of this study are contrary to findings from past studies. Past studies indicated that males and females experience different psychological distress [47,48]. The present study did not find any significant gender difference in psychological distress. Findings indicated that males and females experience a similar amount of psychological distress. A possible explanation for this might be that law enforcement [25] and the prohibition of accessibility by the country’s institutions [20] hinder these emerging adults from overly immersing themselves in the IP materials to the extent that it affects their psychological health. Hence, neither males nor females report differences in their psychological distress.

Furthermore, it is interesting to note the exception of the subscales of salience and tolerance in the overall problematic pornography consumption scale that was significantly correlated with psychological distress. These results further support the idea of cultural differences [48,67] in the Malaysian context. Emerging adults in Malaysia live in a conservative society that sees the possibility of the behaviour of IPU receiving significant disapproval from the public. Thus, it is less likely to find Malaysian emerging adults using IPU, particularly in their daily lives (salience) or gradually increasing their watching time while looking out for more diverse IP (tolerance).

Finally, the gendered nature of problematic IPU has filled a notable knowledge gap for the interaction effects of problematic IPU and gender on psychological distress. The results implied that females who reported their IPU at problematic levels would experience more psychological distress. This contrasts with the results of problematic Instagram use that indicated significant psychological distress among males [58]. However, there are several possible explanations for this result. Firstly, females with problematic IPU may be more vulnerable to psychopathological symptoms such as loneliness and anxiety. They are more likely to score higher on psychopathological symptoms [58]. Secondly, IP production is a male-dominated industry that often presents sexist content appealing to most male audiences’ tastes [68]. Often, the scripts and scenes in IP are sexually objectifying, where females’ bodies serve as the source of males’ pleasure-seeking [69]. Hence, the devaluation of females portrayed in the scenes can cause females to be more susceptible to psychological distress. The result also further depicts that the sexual script persists among males and females where the cultural scenarios have greater tolerance for males’ engagement in IPU. Taken together, it is speculated that the threshold for women to experience psychological distress is low, mainly when they report a higher IPU. Therefore, even when males and females were at the same level of problematic IPU, women were prone to experiencing greater psychological distress.

### 5.1. Limitations and Recommendations for Future Research

Findings generated from the present study should be viewed by considering several limitations. Firstly, most of the participants were Chinese in the race, which may not accurately represent the ethnic ratio in Malaysia. Hence, future studies may consider recruiting respondents following the proportion of ethnicities in Malaysian society. Secondly, the quantitative nature of the present study may not be able to provide an in-depth understanding of individual differences in their problematic IPU, given that the research on this topic is still at an infancy stage. Thus, a mixed-method research design or online photovoice (OPV), an innovative new qualitative method [70], could be considered, which could provide a more comprehensive and holistic outlook on this topic. Thirdly, the cross-sectional design of the present study also limits the cause-and-effect determination of the variables as the literature suggests that the relationship between IPU and psychological distress can be bidirectional [71]. Therefore, future researchers could consider employing longitudinal studies to explain in detail the cause-and-effect of the variables. Finally, the Kessler Psychological Distress Scale is merely a general distress scale on which participants may not only report the distress experienced from IPU. Therefore, an instrument that solely examines psychological distress directly related to IPU should be considered.

### 5.2. Implications

The results have made several noteworthy contributions to the study of IPU in Malaysia, where the sampling focused on emerging adults that mark individuals’ unique developmental transition periods. Moreover, the present study also provides preliminary evidence of high IPU even when it is taboo for Malaysians. Males still tend to report more problematic IPU at a low level. Taken together, the results have filled the knowledge gap and enriched the public debates of the IPU, specifically in Malaysia. As a result, policymakers can be better informed as to how the intervention programmes dealing with the issue of IPU can focus more on female emerging adults. On top of that, the present study results can be a reference for the implementation of sexual reproductive health programmes concerning pornography usage. As such, the topic of media literacy (e.g., pornography production and realism) should be introduced to youngsters to reduce the negative effect of pornography use and its impact on their psychological distress.

## 6. Conclusions

In conclusion, problematic IPU is significantly correlated with psychological distress and males are more likely to experience problematic IPU than females. Meanwhile, the present study also provides preliminary statistical support for the moderating role of gender in the association between problematic IPU and psychological distress among emerging adults in Malaysia. Females tend to be more affected by such relationships. Taken together, the results of the study add to the limited knowledge of self-reported IPU in the still understudied area of psychological distress among emerging adults in Malaysia.

## Figures and Tables

**Table 1:** Demographic information of the participants (*n* = 144).

	*n*	%	Mean	SD	Min.	Max.
Age			21.41	3.49	18	29
Gender						
Male	91	63.2				
Female	53	36.8				
Ethnicity						
Malays	28	19.4				
Chinese	98	68.1				
Indians	16	11.1				
Others	2	1.4				
Employment Status						
Students	98	68.1				
Full-time employed	34	23.6				
Unemployed	12	8.3				
Relationship Status						
Single	84	58.7				
In-relationship	43	30.1				
Married	16	11.2				

Note: SD = Standard deviation.

**Table 2:** Gender differences in problematic IPU (*n* = 144).

Variable	Total		Male		Female		*t*	*p*
	Mean	SD	Mean	SD	Mean	SD		
Salience	2.96	1.51	3.30	1.54	2.38	1.27	3.83	<0.001
Mood Modification	3.10	1.68	3.42	1.67	2.54	1.55	3.16	0.002
Conflict	2.31	1.35	2.55	1.42	1.91	1.15	2.93	0.004
Tolerance	2.50	1.47	2.69	1.50	2.19	1.36	2.05	0.043
Relapse	2.86	1.57	3.23	1.54	2.21	1.42	4.01	<0.001
Withdrawal	2.24	1.42	2.40	1.45	1.96	1.35	1.81	0.073
Problematic Pornography Use	2.66	1.28	2.93	1.25	2.20	1.22	3.42	0.001

Note: SD = Standard Deviation.

**Table 3:** Matric correlation between variables (*n* = 144).

	1	2	3	4	5	6	7	8
1. Salience	1							
2. Mood modification	0.81 ***	1						
3. Conflict	0.44 ***	0.47 ***	1					
4. Tolerance	0.73 ***	0.72 ***	0.60 ***	1				
5. Relapse	0.78 ***	0.71 ***	0.53 ***	0.77 ***	1			
6. Withdrawal	0.78 ***	0.75 ***	0.51 ***	0.78 ***	0.75 ***	1		
7. Problematic IPU	0.89 ***	0.88 ***	0.68 ***	0.89 ***	0.89 ***	0.89 ***	1	
8. Psychological distress	0.13	0.17 *	0.28 ***	0.12	0.17 *	0.19 *	0.20 **	1

Note: *** *p* < 0.001; ** *p* < 0.01; * *p* < 0.05.

## Data Availability

The datasets generated during and/or analyzed in this study are available on request from the first corresponding author.
